# Soluble HMGB1 Is a Novel Adipokine Stimulating IL-6 Secretion through RAGE Receptor in SW872 Preadipocyte Cell Line: Contribution to Chronic Inflammation in Fat Tissue

**DOI:** 10.1371/journal.pone.0076039

**Published:** 2013-09-20

**Authors:** Brice Nativel, Mery Marimoutou, Vincent G. Thon-Hon, Manoj Kumar Gunasekaran, Jessica Andries, Giovédie Stanislas, Cynthia Planesse, Christine Robert Da Silva, Maya Césari, Thomas Iwema, Philippe Gasque, Wildriss Viranaicken

**Affiliations:** 1 Groupe de Recherche Immunopathologie et maladies Infectieuses, Université de La Réunion, Réunion, France; 2 Groupe d’Etude sur l’Inflammation Chronique et l’Obésité, Université de La Réunion, Réunion, France; University of Minnesota - Twin Cities, United States of America

## Abstract

Low-grade inflammation (LGI) is a central phenomenon in the genesis of obesity and insulin-resistance characterized by IL-6 in human serum. Whereas this LGI was initially thought to be mainly attributed to macrophage activation, it is now known that pre-adipocytes and adipocytes secrete several adipokines including IL-6 and participate to LGI and associated pathologies. In macrophages, HMGB1 is a nuclear yet secreted protein and acts as a cytokine to drive the production of inflammatory molecules through RAGE and TLR2/4. In this paper we tested the secretion of HMGB1 and the auto- and paracrine contribution to fat inflammation using the human preadipocyte cell line SW872 as a model. We showed that 1) human SW872 secreted actively HMGB1, 2) IL-6 production was positively linked to high levels of secreted HMGB1, 3) recombinant HMGB1 boosted IL-6 expression and this effect was mediated by the receptor RAGE and did not involve TLR2 or TLR4. These results suggest that HMGB1 is a major adipokine contributing to LGI implementation and maintenance, and can be considered as a target to develop news therapeutics in LGI associated pathologies such as obesity and type II diabetes.

## Introduction

HMGB1 is a non-histone nuclear protein which is a highly conserved, ubiquitous and is comprised of 215 amino acids that are organized in two globular DNA-binding domains, box A and box B, and with an acidic C-terminal tail [1], [2]. HMGB1 can bind DNA, helps chromosome architecture organization and regulates transcription of genes. Outside the cell, HMGB1 can function as a chemokine or an alarmin to activate the immune system and mediate a wide range of physiological and pathological responses including autoimmunity, cancer, trauma, hemorrhagic shock and ischemia-reperfusion injury [3]. HMGB1 can be released in two conditions: an active secretion by immune cells or a passive secretion initiated by cellular damage like cell death [4]. The shuttling of HMGB1 between the nucleus and the cytoplasm is dependent of post-translational modifications that influence its traffic [5].

Once in the extracellular space, HMGB1 interacts with the receptor for advanced glycation end products (RAGE) and/or members of the Toll-like family of receptors (TLRs) including TLR2 and TLR4. Activation of these receptors results in the activation of NF-κB, which influences the production of pro-inflammatory cytokines [3]. Moreover, activation of NF-κB induces the expression of HMGB1 receptors, and increases secretion of HMGB1 inducing a positive feedback loop of HMGB1-mediated inflammatory response [6].

The role of HMGB1 on macrophages is clearly described and stimulation of macrophages with HMGB1 induces a production of pro-inflammatory cytokines [7] which can lead to an increase of adipose tissue inflammation and insulin resistance.

Obese adipose tissue is characterized by low grade inflammation (LGI) [8] that is characterized by robust secretion of pro-inflammatory cytokines including IL-6 and active recruitment of leukocytes, mainly macrophages in the affected tissues [9]. These cytokines are known to be involved in insulin desensitization phenomenon which could contribute to obesity and type 2 diabetes [10].

However long before the onset of obesity and macrophages infiltration, adipose tissue is able to implement and maintain LGI. Indeed it has been recognized that adipocytes also demonstrate significant inflammatory properties like IL-6 or TNFα production [11], [12]. IL-6 expression in adipocytes is governed by an autocrine positive feedback loop and is upregulated by insulin. It has been shown that IL-6 upregulation in adipocytes leads to insulin resistance by activating SOCS3 in target cells like hepatocytes [13], [14], [15] .

Moreover, pre-adipocytes contribute significantly to inflammation of the adipose tissue in obesity [16], [17], [18]. It has been demonstrated that pre-adipocytes can function as macrophage-like cells and may play a direct role in the inflammation response and obesity [19], [20]. Expression profile of pro-inflammatory factors differs between adipocytes and pre-adipocytes and they may play different, but yet, complementary roles in inflammation of adipose tissues [18]. In addition, adipocytes can control the proliferation of pre-adipocytes [21] which accumulate thereby contributing further to inflammation of the adipose tissue. Pre-adipocytes and adipocytes produced mostly IL-8 and IL-6 pro-inflammatory cytokines and less TNF-α [22] which is mostly produced by macrophages. All of these observations show a central role of pre-adipocytes and adipocytes in the inflammatory status and LGI of adipose tissue mainly mediated by IL-6 secretion and its implication in insulin resistance.

A number of studies have demonstrated an autocrine and paracrine effect of HMGB1 on macrophages which can potent LGI. However, it is not known whether preadipocytes and adipocytes may secrete and respond to HMGB1. There is currently no published report regarding HMGB1 expression and functions in fat cells and possible links to LGI in obesity. In this study we investigated the implication of HMGB1 in the regulation of inflammation in preadipocytes using the human preadipocyte cell line SW872 as a model. We showed that HMGB1 is released actively by SW872 and that extracellular HMGB1 contributes to IL-6 secretion through RAGE. These results suggest that HMGB1 and associated-signaling pathways could be used as targets for the development of new therapeutics against pathologies related to adipose tissue inflammation, insulin resistance and diabetes.

## Materials and Methods

### Drugs and reagents

Except when indicated, all drugs and reagents were from Sigma Aldrich (Sigma-Aldrich, France).

### Production of recombinant HMGB1 (rHMGB1)

The full length version of human HMGB1 (clone image 5268698, accession number BC066889) was modified by directed mutagenesis to remove the endogenous NdeI restriction site using 5′-AGGCAAAATGTCATCTTATGCATTTTTTGTG-3′ and 5′-CACAAAAAATGCATAAGATGACATTTTGCCT-3′. It was then amplified by PCR using primers 5′-AACCATGGGCAAAGGAGATCCTAA-3′ and 5′-TTTGAATTCTTGCTGCCGCGCGGCACCAGTTCATCATCATCATCTTCTT-3′ and subsequently cloned in pET28 vector using NcoI and EcoRI sites. This lead to a sequence encoding HMGB1 – thrombin site – C-terminal his-tag. After transformation of BL21-codon plus *E. Coli* bacteria (Agilent), culture and induction were done as described [23]. Cells were harvested at 3,000 g for 15 min at 4°C. All subsequent steps were done at 4°C. The pellet was resuspended in 10 mL of 25 mmol.L^−1^ Hepes (PAN Biotech, Germany). Cells were lyzed by sonification (Misonix ultrasonic liquid processor, France) at 80% of amplitude for 2 min with 10 sec of burst and 10 sec of pause in the ice. Lysate was centrifuged at 15,000 g for 10 min at 4°C, the supernatant was submitted to the action of DNAse I at 80 U/mL for 30 min at 25°C, then diluted to a final volume of 10 mL and mixed gently with 0.5 mL of agarose Ni-NTA (Qiagen, France). After 30 min, the column was washed with 10 mL of 25 mmol.L^−1^ Hepes, 500 mmol.L^−1^ NaCl, then with 10 mL of 25 mmol.L^−1^ Hepes, 20 mmol.L^−1^ imidazole, then with 25 mL of 25 mmol.L^−1^ Hepes, 0.1% (v/v) Triton X-114, 20 mmol.L^−1^ imidazole, then a final washing step with 10 mL of 25 mmol.L^−1^ Hepes, 20 mmol.L^−1^ Imidazole. HMGB1-His was eluted with 5 mL of 300 mmol.L^−1^ imidazole in 25 mmol.L^−1^ Hepes. The elute (1 mL) was dialyzed overnight at 4°C against 25 mmol.L^−1^ Hepes. Levels of LPS and DNA in the preparation were evaluated respectively by LAL assay (HyCult Biotechnology, Uden, The Netherlands) and ethidium bromide (EtBr) coloration of DNA after gel electrophoresis. Level of LPS was under 1ng.L^−1^ and no DNA was detected by EtBr coloration. Biochemical evaluation of rHMGB1 was conducted by SDS-PAGE with coomassie-blue staining and western blot (Figure S4A).

### Cell culture and treatment

SW872 liposarcoma cells line (ATCC, HTB-92) were cultured at 37°C with 5% CO_2_ in DMEM medium, supplemented with 10% heat inactivated fetal bovine serum and 2 mmol.L^−1^ L-Glutamine, 1 mmol.L^−1^ sodium pyruvate, 100 U.mL^−1^ of penicillin, 0.1 mg.mL^−1^ of streptomycin and 0.5 µg.mL^−1^ of fungizone (PAN Biotech, Germany). All experiments were done between passage 2 and 8 at 80% of confluence. For treatment, the media was replaced by fresh complete media (see above) containing drugs or rHMGB1 for 5 h or indicated time. Cells were harvested and kept as frozen pellet for further protein or mRNA analysis. The media were centrifuged for 5 min at 800 g and supernatants were kept at −20°C until use for quantification by ELISA or Dot-Blot.

### RNA interference and retroviral gene transfert

29mer shRNA constructs (TR316576) in retroviral untagged pRS vector, against HMGB1 were purchased from Origene (Clinsciences, France). Retroviral particule were produced in PT-67 packaging cell lines as described by manufacturer’s instructions (Clontech, Ozyme, France). SW872 were stably infected using the tissue cell supernantant of transfected PT67 cells as before using puromycin (1 µg.mL^−1^) as selection agent for twenty days [24].

### Blocking antibody assay

SW872 cells were treated with rHMGB1 (1 µg.mL^−1^) and incubated with control or blocking antibodies for 5 h at 1∶20 dilution. Blocking antibodies against TLR2 (clone T2.5, 5 µg.mL^−1^) and TLR4 (clone HTA125, 5 µg.mL^−1^) [25], [26] were purchased from HyCult Biotechnology (Uden, The Netherlands). Blocking polyclonal antibody against RAGE used at 1∶20 dilution (goat anti-RAGE, AB5484) was purchased from Chemicon International Inc (Temecula, CA, USA). Blocking rabbit monoclonal anti-HMGB1 used at 1∶20 dilution (clone D3E5, 6893) was obtained from Cell Signaling Technology (Ozyme, Saint Quentin, France). Blocking activity of antibody against RAGE and HMGB1 for cytokine activity of HMGB1, were validated in RAW-BLUE cells using rHMGB1 at 1 µg.mL^−1^ (Invivogen, France).

### Lysate preparation and Western Blot analysis

SW872 cells were harvested after two washes with PBS and pelleted at 800 g for 5 min at 4°C. The pellets were processed for subcellular fractionation using the NE-PER according to manufacturer’s instruction (Thermo Scientific, France). Protein extracts (cytoplasmic or nuclear fractions) were separated by SDS-PAGE and transferred to nitrocellulose membranes (Hybond-C extra, GE Healthcare, France). The Western blot was performed as described [27]. Monoclonal anti-HMGB1 (clone 2F6 Sigma), monoclonal anti-β-actin (clone C4, Santa Cruz Biotechnology), monoclonal anti-lamin B (Oncogene, Cambridge, MA, USA) were used as primary antibodies at 1∶1000 dilution. Anti-rabbit immunoglobulin-horseradish peroxidase or anti-mouse immunoglobulin-horseradish peroxidase conjugates (Vector, France) were used as secondary antibodies and diluted at 1∶2000. All antibodies were diluted in TRIS buffer with 0.1% Tween and 5% (w/v) low fat milk. Blots were revealed with ECL detection reagents (GE Healthcare).

### ELISA quantification and Dot-Blot analysis

Secretion of cytokines (IL-6, TNF-α) was measured by ELISA kit (eBiosciences, Austria) according to manufacturer’s instructions. Of note, no exogenous HMGB1, LPS and IL-6 were detected in commercial cell culture media or complete media by Dot-blot, LAL-assay and ELISA, respectively (data not shown). Secretion of HMGB1 was measured by ELISA kit from IBL international according to manufacturer’s instructions (IBL, Germany). 100 µL of SW872 cell culture supernatant were spotted on nitrocellulose membrane (Hybon-C extra, GE Healthcare, France) with a Dot-blot apparatus (Biorad). Membranes were processed as for the Western-blot technique.

### RNA isolation and RT-PCR

Total RNA was extracted with TRIzol reagent (Invitrogen, Carlsbad, USA) according to the manufacturer’s specifications. The reverse transcription (RT) was done with 2 µg of total extracted RNA and 14.5 µL of RT mix containing: 6 µL of 5X RT buffer (Invitrogen), 1.5 µL of 0.1 mol.L^−1^ DTT (Invitrogen), 3 µL of 10 mmol.L^−1^ dNTP mix (Promega, Madison), 2.5 µL of 5 U.mL^−1^ of random hexamers (pdN6), 1.5 µL of 40 U.µL^−1^ RNAse Inhibitor (Promega). After a denaturation at 65°C for 5 min, 2 µL of RTase 200 U.µL^−1^ (Invitrogen) were distributed. The RT steps are the followings, 37°C for 1 h, 95°C for 5 min. cDNAs were then kept at 4°C. The PCR was done in a final volume of 20 µL containing 4 µL of 5X PCR buffer (Promega), 2 µL of 25 mmol.L^−1^ MgCl_2_ (Promega), 0.4 µL of 10 mmol.L^−1^ dNTP mix, 0.1 µL of 5U.µL^−1^ Taq polymerase (Promega), 11.5 µL of water DNase RNase free, 1 µL of cDNA and 1 µL of 10 mmol.L^−1^ primer mix (Table 1). 30 cycles of PCR were done with a pre-denaturation at 95°C for 2 min, 30 cycles of PCR (denaturation at 95°C for 30 sec, annealing at 60°C for 30 sec, extension at 72°C for 45 sec). Final extension was carried out at 72°C for 5 min, and samples were then kept at 4°C. The size of PCR products was checked on 1.2% agarose gel electrophoresis and visualized with EtBr. GAPDH was used as a housekeeping gene.

**Table 1:** List of primers used in this study for RT-PCR on SW872 RNA.

Gene	Genre	Genbank	Primers	Sequences	Amplicon (pb)
GAPDH	human	NM_002046.4	Forward	5'- GCCTTCTCCATGGTGGTGAA -3'	151
			Reverse	5'- GCACCGTCAAGGCTGAGAGAAC -3'	
TLR2	human	NM_003264.3	Forward	5'- GCCTCTCCAAGGAAGAATCC -3'	329
			Reverse	5'- AGAAGAAAGGGGCTTGAACC -3'	
TLR4	human	NM_138557.2	Forward	5'- ACCCTTTAGCCCAGAACTGC -3'	227
			Reverse	5'- GTCTCACGCAGGAGAGCCAG -3'	
RAGE	human	NM_001136.4	Forward	5'- GCCAGAAGGTGGAGCAGTAG -3'	357
			Reverse	5'- AAGATGACCCCAATGAGCAG -3'	
HMGB1	human	NM_002128.4	Forward	5'- CCAAAGGGGAGACAAAAAAAG -3'	193
			Reverse	5'- TCATAAGGCTGCTTGTCATCT -3'	
IL-6	human	NM_000600.3	Forward	5'- ACCCCCAGGAGAAGATTCCA -3'	162
			Reverse	5'- GCCTCTTTGCTGCTTTCACA -3'	
MCP1	human	NM_002982.3	Forward	5'- CAATAGGAAGATCTCAGTGC -3'	188
			Reverse	5'- GTGTTCAAGTCTTCGGAGTT -3'	
TNF-α	human	NM_000594.3	Forward	5'- TCCAGGCGGTGCTTGTTCCT -3'	194
			Reverse	5'- TGGGCTACAGGCTTGTCACTCG -3'	

### Immunofluorescence

SW872 cells were grown on glass coverslips, washed twice with PBS and then fixed for 5 min in fixation buffer (5% formaldehyde v/v and 5% glutaraldehyde v/v in PBS) and washed twice with PBS. Coverslips were conserved at −20°C. Cells were incubated in the following primary antibody, anti-HMGB1 (Clone 2F6, Sigma) 1∶200 dilution (2.5 µg.mL^−1^) in PBS-BSA 1% (w/v) and incubated with the secondary antibody Alexa Fluor 594-conjugated anti-mouse IgG (1∶1000 dilution). Nuclei were stained with DAPI (Sigma, D9542) at a final concentration of 0.1 ng.mL^−1^). Coverslips were mounted in Vectashield (Vector Labs, Clinisciences, France), and fluorescence was observed using a Nikon Eclipse E2000-U microscope (Nikon, Tokyo, Japan). Images were obtained using the Nikon Digital sight PS-U1 camera system and the imaging software NIS-Element AR (Nikon, Tokyo, Japan).

### Flow cytometry

Cells were harvested after two washes with PBS, then pelleted at 800 g for 5 min. Cells were incubated with 5% (w/v) BSA in PBS for 10 min at room temperature under agitation. Primary and secondary antibodies were diluted in PBS-BSA and incubated at room temperature with agitation for 45 min. Primary antibodies were anti-TLR2 (clone T2.5, 25 µg.mL^−1^, HyCult Biotechnology), anti-TLR4 directly conjugated with R-Phycoerythrin (clone HTA125, 25 µg.mL^−1^ HyCult Biotechnology) or anti-RAGE (polyclonal antibody, Chemicon international) at 1∶100 dilution. When required, we used anti-goat or anti-mouse conjugated to R-Phycoerythrin (polyclonal antibody, Southern Biotech) at 1∶500 dilution as secondary antibodies. After two washes with PBS, cells were harvested in PBS. For each experiment 10000 cells were counted and were analyzed by flow cytometry (Beckman Coulter FC-500-MPL Flow Cytometer, MXP acquisition software and CXP analysis software, version 2.2).

### Statistical analysis

All values are expressed as mean +/− SD and are representative at least of 3 independent experiments. Comparisons between different treatments have been analyzed by a one-way ANOVA test. Values of p<0.05 were considered statistically significant for a post-hoc Tukey-Kramer test in order to compare treated *versus* non-treated. All statistical tests were done using the software Graph-Pad Prism version 5.01 (San Diego, CA, USA). Degrees of significance are indicated in the figure captions as follow: * p<0.05; ** p<0.01; *** p<0.001, ns  =  not significant.

## Results

### 1. SW872 as a preadipocyte model

In this study we wanted to test the secretion of HMGB1 and the contribution to fat inflammation of preadipocyte. We used the human liposarcoma cell line SW872 as a model. We first tested the phenotype of SW872 considered as a preadipocyte model. Using RT-PCR we found expression of preadipocyte markers Pref1, GATA2 and AEBP in SW872 as in 3T3L1 preadipocyte (Table S1, Figure S1). We were not able to detect adipocyte markers FABP4, C/EBPα, PPARγ, KLF15, transcripts and the presence of lipids droplets by Oil Red O staining at any time of our experimental settings (data not shown). All of these observations argue in favor of preadipocyte status for SW872 cell line.

### 2. HMGB1 localization in the human preadipocyte cell line SW872

**Figure 1:**
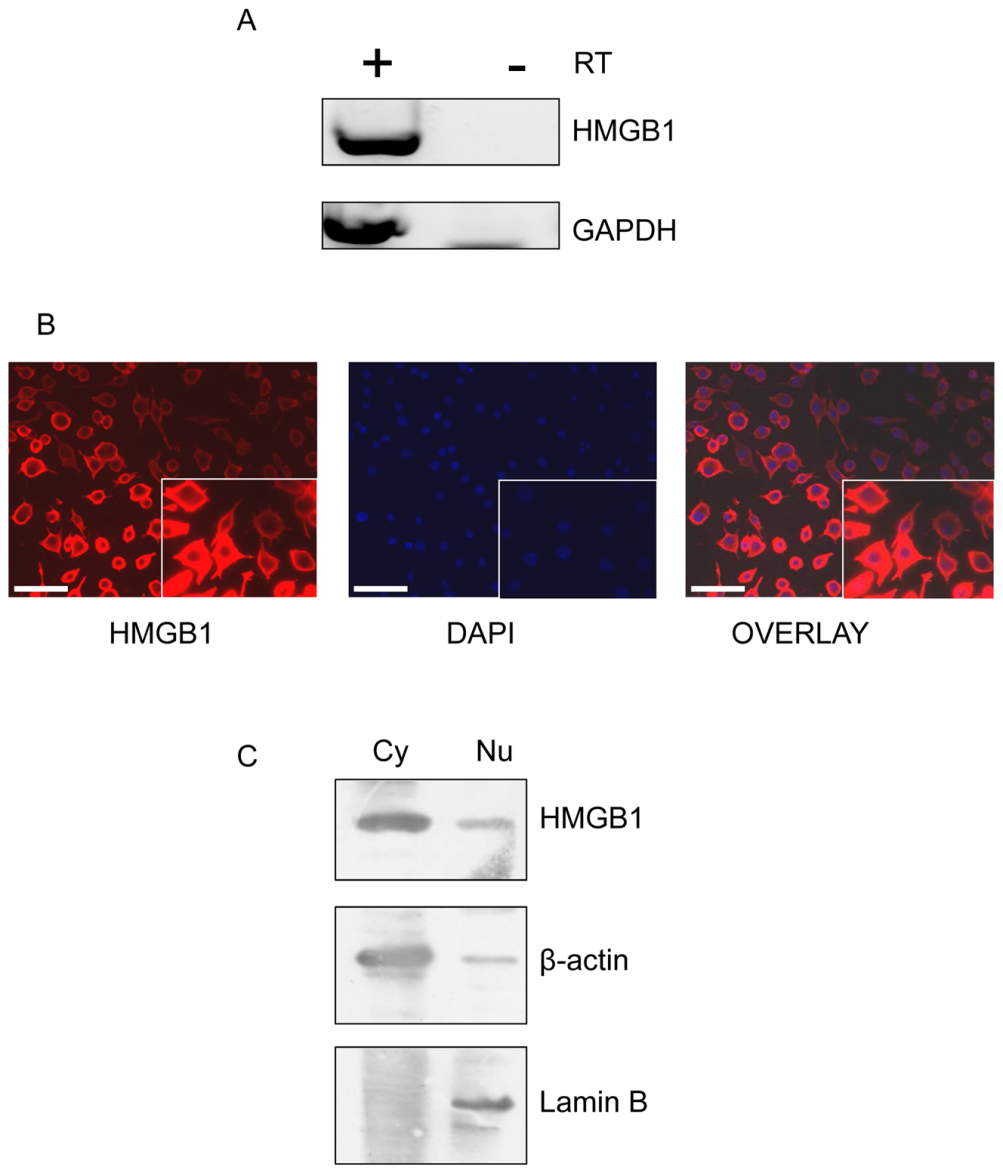
HMGB1 expression and localization in the human preadipocyte cell line SW872. A) Expression of HMGB1 by RT-PCR from total RNA obtained from SW872 cell after 12h of culture, with (+) and without (−) Reverse Transcriptase (RT). B) Detection of HMGB1 protein in different cell fraction by Western blot analysis. β-actin is a cytoplasmic protein (Cy) and lamin B a nuclear protein (Nu). C) Immunofluorescence of HMGB1 in SW872 cells (X 200). Cells were incubated with mouse anti-HMGB1 primary antibody (1:200), Alexa Fluor 594 linked secondary antibody (1∶1000) and DAPI. Scale bar indicated 50 µm.

We analyzed the basal expression of HMGB1 in the human preadipocyte SW872 cells by RT-PCR. The transcript of HMGB1 was detected in SW872 cells in basal culture conditions (Figure 1A). We next investigated the cellular localization of HMGB1 in SW872 cells by immunofluorescence. We found that 100% of SW872 cells expressed HMGB1 and with an immunostaining mainly localized to the cytoplasm (Figure 1B). To confirm that SW872 cells predominantly expressed cytoplasmic HMGB1, we performed Western blot of cellular fractions of cytoplasm (Cy) and nucleus (Nu). The proteins β-actin and lamin B were used as internal controls to assess the enrichment for cytoplasmic and nuclear proteins, respectively. High levels of cytoplasmic and low amount of nuclear HMGB1 were detected in SW872 cells confirming the immunofluorescence data (Figure 1C). These results suggest that HMGB1 accumulates in the cytoplasm of the human preadipocyte cell line SW872. The same results were observed using the mouse 3T3L1 preadipocyte cell line (Figure S2).

### 3. Secreted HMGB1 level correlates with IL-6 secretion in SW872 cells

**Figure 2:**
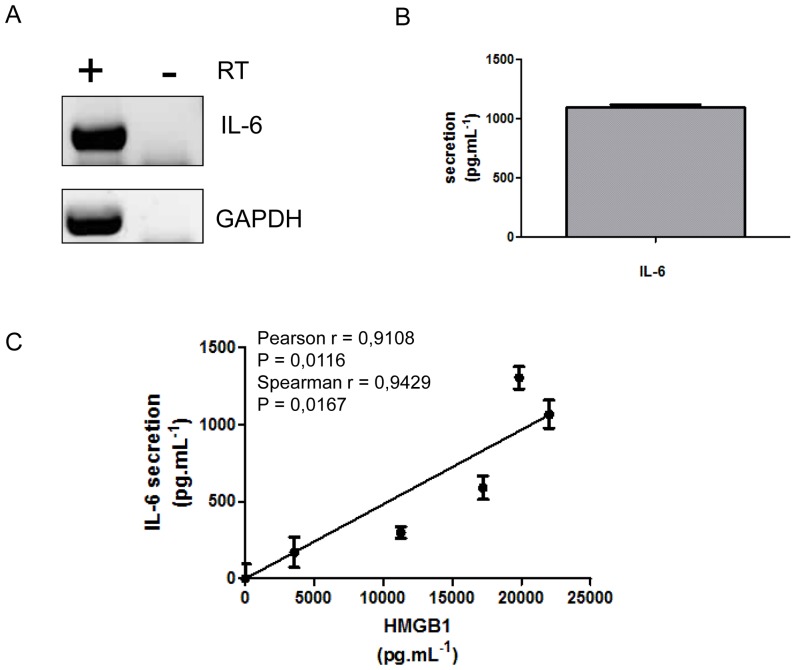
Secreted HMGB1 levels correlate with IL-6 secretion in SW872 cells. A) Expression of IL-6 by RT-PCR from total RNA obtained from SW872 cell after 12 h in culture, with (+) and without (−) Reverse Transcriptase (RT). B) IL-6 secretion by ELISA assay from SW872 supernatant culture 6h after media renewal (expressed in mean ± SD) (n = 3). D) Correlation between IL-6 secretion and HMGB1 release in SW872 cell culture media after renewal of media (n = 3). Pearson's correlation coefficient (r = 0.9108, p = 0.0116) and Spearman's rank correlation coefficient (r = 0.9429, p = 0.0167). All values are expressed as means +/− SD.

It was previously shown that high levels of HMGB1 in the cytoplasm were typically associated with its active secretion [28]. We first tested the release of HMGB1 by SW872 cells by Dot blot from tissue cell supernatant (TCS). HMGB1 was released as soon as 30 min after media renewal and the level of secreted HMGB1 increased continuously (Figure S3A). We did not observe SW872 cell necrosis during this experiment as addressed by the LDH assay (Figure S3B). Hence, HMGB1 secretion was not due to passive release. All these observations also occurred in 3T3L1 preadipocyte cell lines (Figure S3C). We next used RT-PCR and ELISA to show that IL-6 is expressed (Figure 2A) and released (1093.63 pg.mL^−1^ ±24.57) by SW872 cells in normal cell culture conditions (Figure 2B).

In order to investigate a potential correlation between HMGB1 and IL-6 secretion in SW872 cells we evaluated the release of IL-6 and HMGB1 by ELISA after media renewal. We further demonstrated a significant positive relationship between secreted HMGB1 levels and IL-6 release (Spearman’s rank correlation coefficient: r = 0.9429 and p<0.05; Pearson’s correlation coefficient: r = 0.9108 and p<0.05) (Figure 2C). These results suggest that the expression and release of IL6 correlated with the release of HMGB1 by SW872 cells as in 3T3L1 (Figure S3C).

### 4. Secreted HMGB1 controls the release of IL-6 in SW872 cells

**Figure 3:**
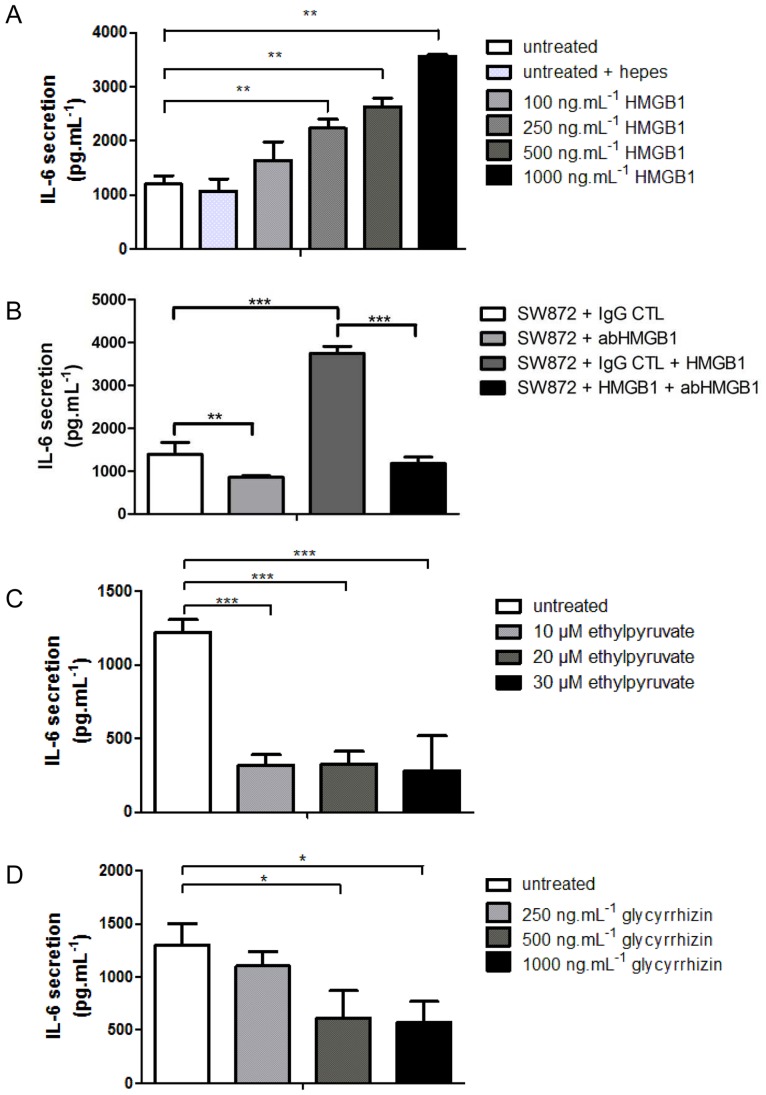
Secreted HMGB1 controls IL-6 production in SW872 cells. IL-6 levels were determined by ELISA from SW872 cells A) treated with increasing concentrations of recombinant HMGB1 for 5 h (n = 4) or B) treated or not with rHMGB1 with a dose of 1 µg.mL^−1^ for 5 h in presence of either an irrelevant control rabbit IgG (CTL) or a rabbit monoclonal anti-HMGB1 (abHMGB1) (n = 4). Detection of IL-6 secretion in SW872 cells treated for 5 h with C) ethyl-pyruvate (n = 3) or D) glycyrrhizin (n = 3). All values are expressed as means +/− SD. Degrees of significance are indicated in the figure captions as follow, * p<0.05, ** p<0.01, *** p<0.001.

We produced a recombinant form of human HMGB1 (rHMGB1) to investigate the direct effects of HMGB1 on SW872 adipocytes (Figure S4A). SW872 cells were stimulated with increasing concentrations of rHMGB1 and levels of secreted IL-6 were quantified by ELISA. Whereas the secretion of IL-6 was not significantly affected after treatment of SW872 cells with 100 ng.mL^−1^ of rHMGB1, higher concentrations of rHMGB1 significantly enhanced the release of IL-6 (p<0.01) and in a dose dependent manner (Figure 3A). IL-6 secretion was not related to rHMGB1 cytotoxicity as cell viability was not affected by all tested dose of rHMGB1 (Figure S4B). Moreover, HMGB1 blocking antibody (abHMGB1) induced a significant reduction of IL-6 secretion in SW872 cells stimulated or not stimulated with rHMGB1 (Figure 3B). These results suggest that IL-6 secretion can be regulated by extracellular HMGB1.

**Figure 4:**
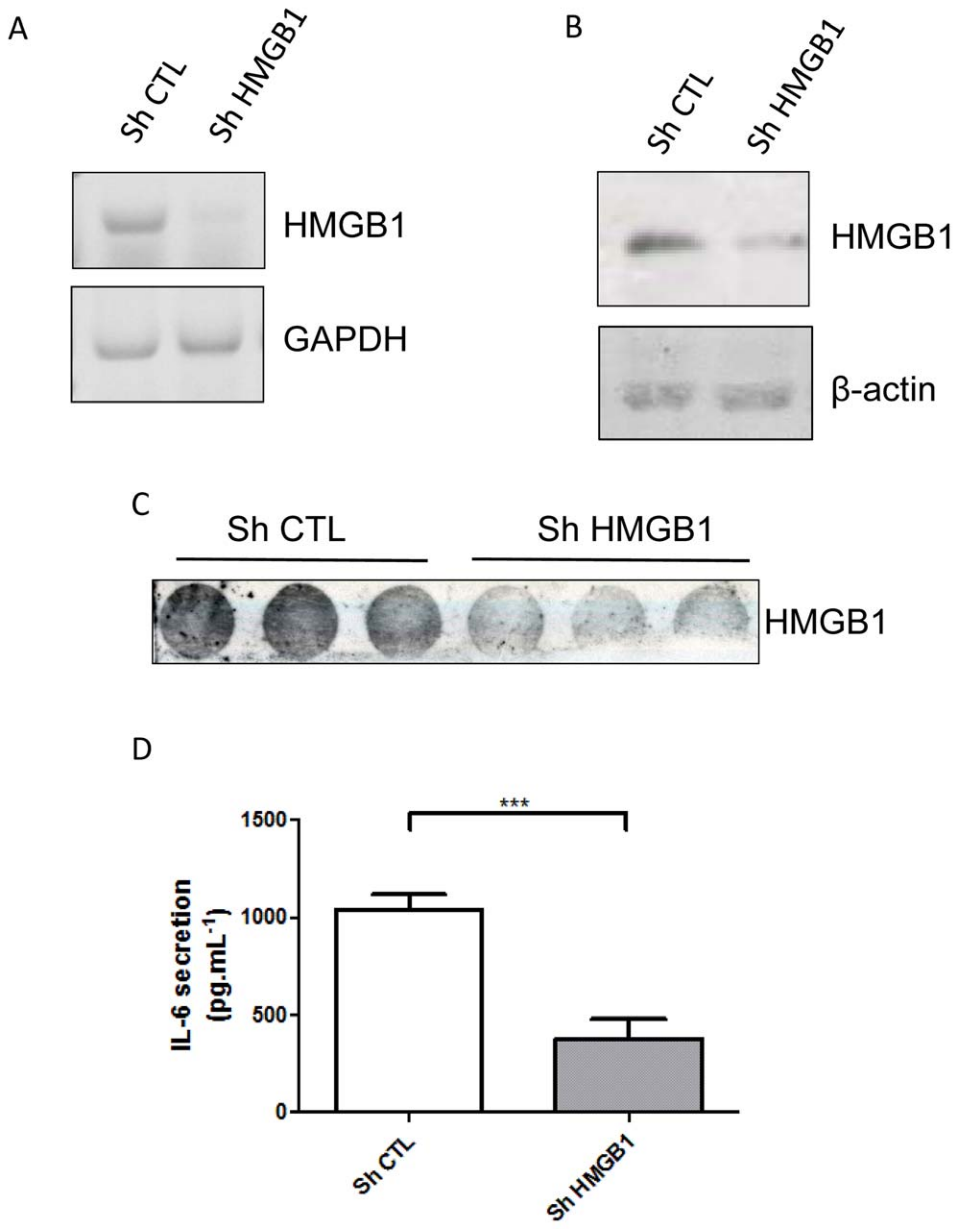
HMGB1 shRNA dowregulates IL6 secretion. HMGB1 mRNA expression or protein expression were assessed respectively by RT-PCR (A) or Western blot (B) after 12h in culture of stably SW872 infected with scrumble shRNA (Sh CTL) or HMGB1 shRNA (sh HMGB1) constructs. C) Detection of IL-6 secretion in stably SW872 infected cells with scramble shRNA (Sh CTL) or HMGB1 shRNA (sh HMGB1) constructs after 5h of media renewal (n = 3). All values are expressed as means +/− SD. Degrees of significance are indicated in the figure captions as follow *** p<0.001.

To further test the contribution of HMGB1 release to IL-6 secretion, cells were treated with two HMGB1 inhibitors, ethyl-pyruvate that blocks the release of HMGB1 (Figure S4C) [29] and glycyrrhizin that interacts with HMGB1 and prevents its binding to its cellular receptors (e.g. TLR2, TLR4, RAGE) [30], [31]. The drug concentrations used herein did not affect cell viability as measured by MTT assay for SW872 (data not shown). The secretion of IL-6 was significantly decreased by 75% (p<0.001) after treatment of SW872 cells with low concentrations of ethyl-pyruvate and in a dose independent manner (319.52 pg.mL^−1^ of IL-6 secretion ± 73.88 after treatment with 10 µmol.L^−1^ of Ethyl-pyruvate, 323.99 pg.mL^−1^ ±88.64 after treatment with 20 µmol.L^−1^ 281.54 pg.mL^−1^±237.78 after treatment with 30 µmol.L^−1^) compared to untreated cells (1220 pg.mL^−1^±88.68) (Figure 3C). Similarly, the release of IL-6 significantly decreased by 55% (p<0.05) after treatment of SW872 cells with 500 ng.mL^−1^ (615.79 pg.mL^−1^ of IL-6 secretion ± 257.01 or 1000 ng.mL^−1^of glycyrrhizin (574.95 pg.mL^−1^±196.24) compared to untreated cells (1300 pg.mL^−1^±202.32) (Figure 3D). To validate our observations we used an shRNA which target human HMGB1 mRNA and efficiently down regulated HMGB1 at mRNA, protein level and in the media (Figure 4A, B and C). HMGB1 down regulation was accompanied by at least 50% reduction (p<0.001) of the release of IL-6 (Figure 4C).

### 5. HMGB1-mediated IL-6 secretion involves the receptor RAGE in SW872 cells

**Figure 5:**
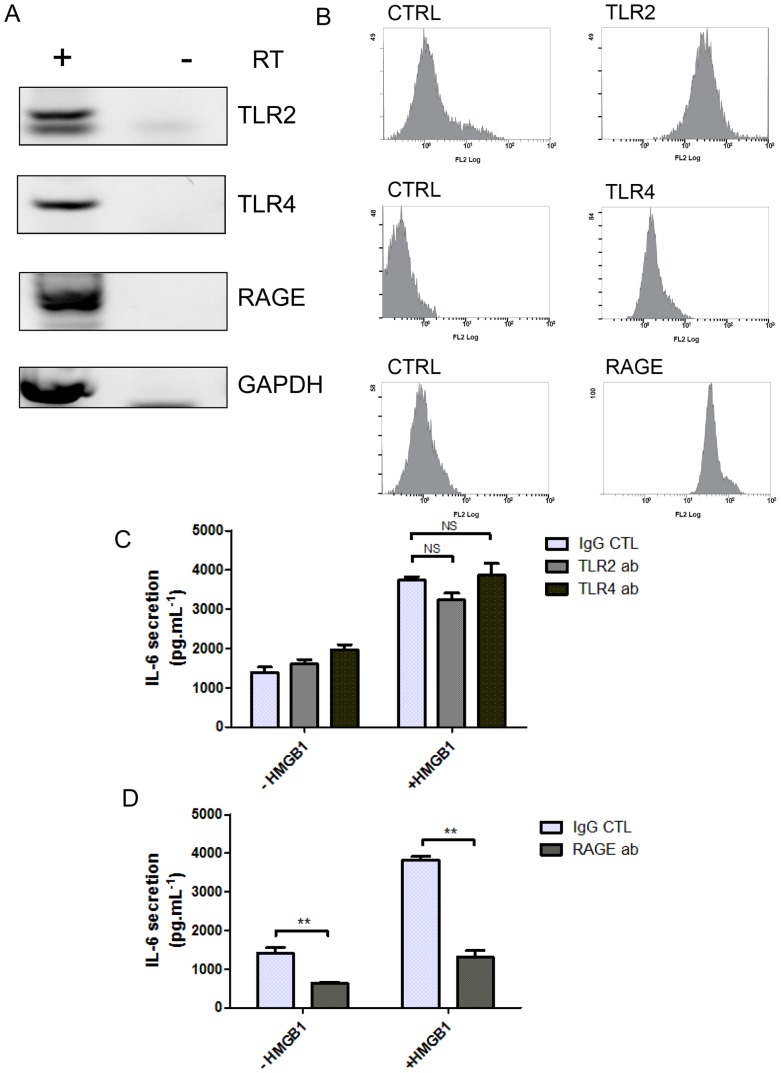
HMGB1 mediates IL-6 release through RAGE. A) TLR2, TLR4, RAGE and HMGB1 mRNA expression was assessed by RT-PCR from total RNA obtained from SW872 cell after 12h in culture, with (+) and without (−) Reverse Transcriptase (RT). B) Cell surface expression of TLR2, TLR4 and RAGE were analyzed by FACS C) SW872 cells were treated with rHMGB1 1 µg.mL^−1^ for 5 h with control mouse IgG (CTL), TLR2 or TLR4 blocking antibody (n = 4). D) SW872 cells were treated as in (C) except that control goat IgG (CTL) or goat polyclonal anti-RAGE were used (n = 4). All values are expressed as means +/− SD and degrees of significance are indicated in the figure captions as follow, * p<0.05, ** p<0.01, *** p<0.001 and ns  =  not significant.

It is well documented that HMGB1 can bind to the cellular receptors TLR2, TLR4 and RAGE [20]. Hence, we first tested the expression of these receptors in SW872 cells using RT-PCR and FACS analysis. TLR2, TLR4 and RAGE mRNAs were detected in SW872 cells (Figure 5A) and all three receptors were detected at the cell surface by FACS (Fig5B). Next, we investigated whether HMGB1-mediated secretion of IL-6 was dependent of these cell surface receptors in SW872 cells using blocking antibodies against TLR2, TLR4 and RAGE. SW872 cells were treated for 5 h with rHMGB1 together with either an irrelevant mouse antibody (IgG control) or blocking antibodies against TLR2, TLR4 or RAGE as previously described [25]. Whereas TLR2 and TLR4 blocking antibodies had no significant effects on IL-6 secretion induced by rHMGB1 (Figure 5C), RAGE blocking antibody significantly decreased IL-6 secretion by 3 fold (p<0.001) (85.10% of IL-6 secretion ± 12.53%) compared to cells stimulated with rHMGB1 and incubated with irrelevant IgG control (258.89% of IL-6 secretion ± 8.54) (Figure 5D). This result was confirmed using another antibody against RAGE (rabbit anti-RAGE, 4679S, Cell Signaling Technology) (data not shown). Our results suggest that HMGB1-mediated secretion of IL-6 involved the receptor RAGE expressed by SW872 cells.

### 6. HMGB1 control MCP1 secretion in SW872

**Figure 6:**
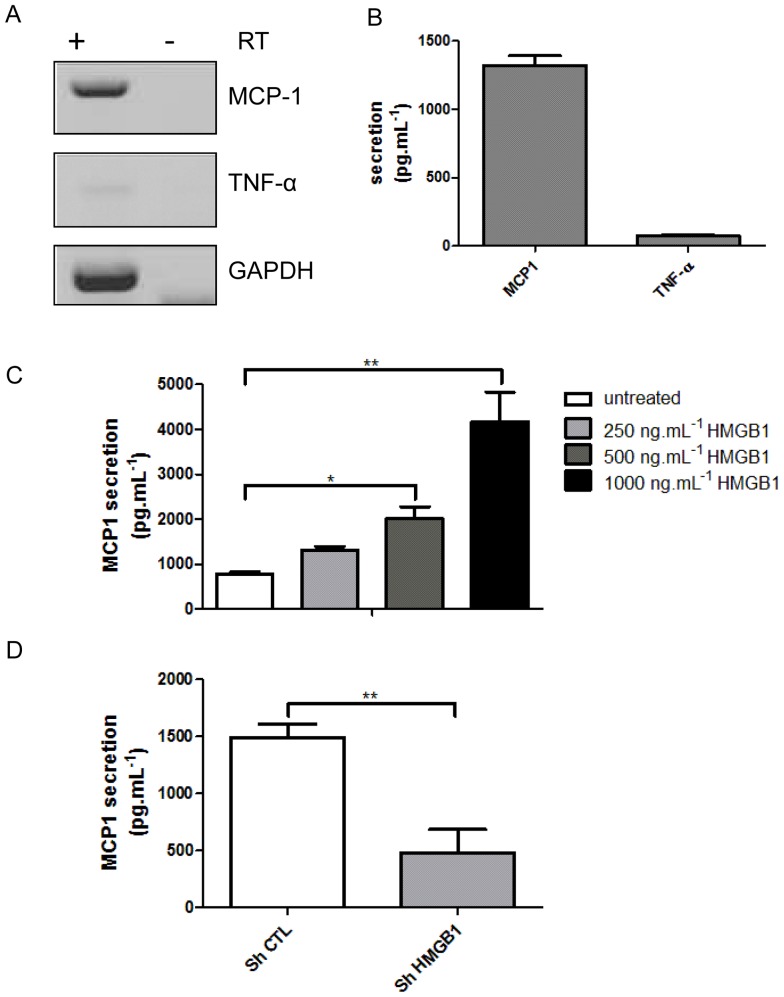
HMGB1 controls MCP1 (CCL2) secretion. A) MCP1 and TNFα mRNA expression were assessed by RT-PCR from total RNA obtained from SW872 cell after 12h in culture, with (+) and without (−) Reverse Transcriptase (RT). B) MCP1 and TNFα secretion by ELISA assay from SW872 supernatant culture 6h after media renewal (n = 6). C) IL-6 levels were determined by ELISA from SW872 cells treated with increasing concentrations of recombinant HMGB1 for 5 h (n = 3). D) Detection of MCP1 secretion in stably infected SW872 with scramble shRNA (Sh CTL) or HMGB1 shRNA (Sh HMGB1) constructs after 5h of media renewal (n = 3). All values are expressed as means +/− SD and degrees of significance are indicated in the figure captions as follow, * p<0.05, ** p<0.01, *** p<0.001.

In LGI other inflammatory markers were also important like TNFα and MCP1 (CCL2). We investigated basal expression of TNFα and MCP1 in our SW872 preadipocyte model, as previously observed [22], [32] high levels of MCP1 was expressed and secreted as addressed by RT-PCR and ELISA respectively (Figure 6 A and B). Low levels of TNFα was detected in basal conditions. The same pattern was found in 3T3L1 preadipocyte by RT-PCR (Figure S5). MCP1 secretion was next monitored in response to treatment of SW872 cells with rHMGB1. rHMGB1 significantly enhanced the release of MCP1 (p<0.01) in a dose dependent manner (Figure 6C).

To validate HMGB1 action, we analyzed MCP1 secretion after treatment of SW872 with plasmid encoding shRNA against HMGB1 as described above for IL6. Down regulation of HMGB1 levels significantly (p<0.01) reduced MCP1 secretion (Figure 6D). All together these observations argue in favor of MCP1 secretion controlled by HMGB1 in preadipocyte.

## Discussion

The secretion of HMGB1 by macrophages is well characterized and essentially through the active release from cytoplasmic stores. Moreover, secreted HMGB1 can induce a signaling cascade leading to the production and release of a plethora of pro-inflammatory molecules and leading to LGI. It is recognized that adipose tissue contributes to LGI but the role of HMGB1 specifically on preadipocytes has remained largely unknown.

To test the hypothesis that HMGB1could participate to LGI in fat tissues, we used the human liposarcoma cell line SW872 as a preadipocyte model [33], [34], [35], [36], [37], [38]. Herein, we showed that HMGB1 accumulated in the cytoplasm of SW872 and was secreted in the cell culture supernatants overtime. This observation is in agreement with a previous study where the authors showed that highly acetylated form of HMGB1 preferentially accumulated in the cytoplasm of monocytes before being secreted [28]. It is generally accepted that a dominant activity of histone acetyl tranferase activity and/or a lower activity of histone deacetylase could contribute to the accumulation of cytoplasmic HMGB1 but this remains to be tested in our cell model.

Interestingly, we found that SW872 cells released constitutively high levels of IL-6 and through a direct action of the endogenous HMGB1. The levels of secreted IL6 correlated positively with the level of HMGB1 and, more importantly, we found that two inhibitors of HMGB1 release impaired IL6 production by SW872. Thus, our data show strong inhibitory activities either with ethyl-pyruvate [29] or glycyrrhizin which interacts specifically to A and B boxes of HMGB1 preventing its binding to its receptors [30]. These two inhibitors interfere with HMGB1 effect on IL-6 secretion. The decrease in IL-6 production mediated by the inhibition of endogenous HMGB1 might suggest an autocrine and paracrine activity of HMGB1 to regulate the secretion of IL-6 in SW872 cells. We observed that IL-6 secretion was less inhibited by glycyrrhizin than by ethyl-pyruvate. This difference might be due to the double action of ethyl-pyruvate which interferes with HMGB1 secretion [29] but also decreases NF-κB DNA binding activity, thus reducing the production of cytokines [39].

When SW872 cells were exposed to rHMGB1, we observed an increased secretion of IL-6 in a dose dependent manner further suggesting that HMGB1 can act in a direct manner on fat cells to promote the release of IL-6.

In a previous study, IL6 secretion was significantly increased in culture medium of adipocytes in presence of LPS [40]. Moreover, LPS was more effective with higher IL-6 secretion in preadipocyte compared to mature adipocytes [16], [17], [18]. It is known that HMGB1 can strongly enhance the cytokine response evoked by LPS [41], [42] and this is to be correlated with the observations that HMGB1 secretion by preadipocyte was not observed in fully mature adipocyte [43]. All together these observations would argue for a more potent inflammatory role of preadipocyte when compared to mature adipocytes.

The strong association between high levels of secreted HMGB1 by SW872 and strong levels of IL-6 is interesting to shed new light on the mechanisms of LGI and possible therapeutic avenues. It has also been shown a strong link between IL-6 serum levels and insulin resistance [8], [15], [44], [45], [46]. Hence, this relation between IL6 and insulin resistance should be extended to propose a central role of HMGB1 in insulin resistance through it action on IL6 secretion. The therapeutic potentials of HMGB1 inhibitors should now be tested in the context of insulin resistance pathologies and experiments along these lines are now highly warranted.

**Figure 7:**
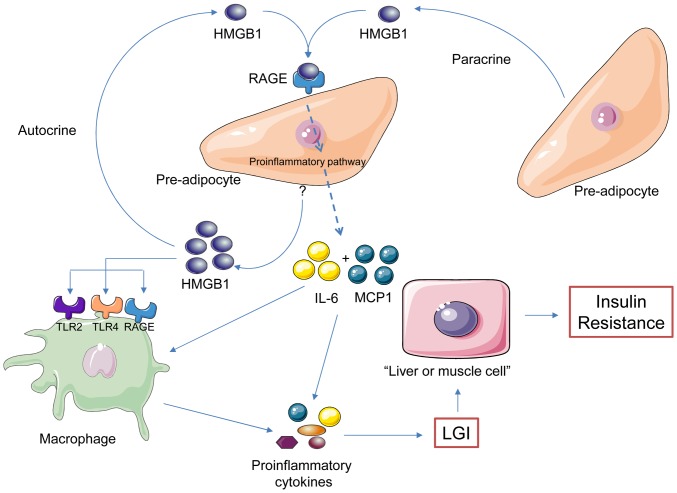
Central role of HMGB1 adipokine in LGI of fat tissue. Model depicting the novel role of HMGB1 as an adipokine involved in fat tissue inflammation, obesity and associated-chronic disorders such as diabetes type II. We found that HMGB1 is secreted and interacts via an autocrine and/or paracrine manner with RAGE on preadipocytes. This could contribute to low grade inflammation (LGI) of the fat tissue with the elevated expression of major pro-inflammatory cytokines such as IL-6 and MCP1. We could further hypothesize that HMGB1 released by preadipocytes can attract, via MCP1, macrophages and leading to activation through TLR2, TLR4 and RAGE. Finally, the pool of proinflammatory factors and recruited immune cells could instigate the LGI and the insulin resistance in liver and muscle cells.

Using our preadipocyte SW872 cell model, we showed that HMGB1 behave as a positive regulator of MCP1 secretion. As MCP1 act as a chemokine which attract macrophages, HMGB1 may contribute to immune cells infiltration in adipose tissue and instigating LGI. The presence of macrophages with potent pro-inflammatory activities and in response to HMGB1 released in the vicinity of the adipocytes may contribute further to insulin resistance (Figure7) [47], [48], [49].

Several key receptors have been implicated in HMGB1 signaling, including RAGE, TLR2 and TLR4. We showed that RAGE, TLR2 and TLR4 are transcribed and expressed at the surface of SW872 cells. However, only RAGE blocking antibody induced a strong inhibition of IL-6 secretion by preadipocytes SW872 stimulated with rHMGB1.

RAGE is known to be implicated in obesity, diabetes and their complications [50]. This implication is mostly related to its capacity to interact with advanced glycation end products (AGE) [51]. AGE can signal through RAGE to activate oxidative stress and inflammation and finally interfere with insulin-signaling pathways in adipocytes to decrease insulin sensitivity [52], [53]. It will be essential to decipher the signaling pathways engaged by HMGB1 binding to RAGE in fat cells and although the role of soluble RAGE (natural antagonist) in this paradigm. Moreover, we will need to address whether AGE and HMGB1 can compete for the same binding site on RAGE and emphasizing possible antagonistic effects yet to be identified. We will need to study the signals downstream of RAGE and perform transcriptomic profiling of SW872 prior to and after HMGB1 stimulation. We used herein a model of human preadipocyte cells and it would be interesting to test the contribution of HMGB1 in primary cultures of human adipose cells and in vivo following the injection of HMGB1 in fat tissue sites.

Our results argue in favor of a central effect of RAGE in fat tissue inflammation. Administration of soluble RAGE or blocking antibody can induce a protective effect against inflammation and diabetes [54], [55] and this could be due to the interference with HMGB1 pro-inflammatory functions. Remarkably, little is known about the distribution of HMGB1 in fat tissues and whether the level of soluble HMGB1 (in blood and fat tissues) is increased in response to obesity. Experiments along these lines are now highly warranted following cohorts of patients and analyzing sera and tissue biopsies for the presence of soluble HMGB1 and possible correlations with elevated levels of proinflammatory cytokines and adipokines.

In summary, our study is the first report ascribing a novel proinflammatory activity of HMGB1 on adipose tissue and particularly in preadipocyte. We can consider that soluble HMGB1 is a novel adipokine secreted by preadipocyte and controlling inflammation through the binding to RAGE receptors [56], [57]. Indeed, our observations showed a direct involvement of HMGB1 in controlling the secretion of IL-6 and MCP1 in adipose tissues (Figure7). Experiments are now highly warranted to decipher the role of HMGB1/RAGE in LGI and numerous associated chronic pathologies.

## Supporting Information

Figure S1 **Preadipocyte markers expression in SW872 and 3T3L1 cell lines.** Expression of pref-1, GATA2, AEBP by RT-PCR from total RNA obtained from SW872 cell after 12 h in culture (A) or from 3T3L1 after 16h in culture, with (+) and without (−) Reverse Transcriptase (RT). GAPDH served as housekeeping gene.
(TIF)

Figure S2 **HMGB1 expression and localization in the human preadipocyte cell line 3T3L1.** A) Expression of HMGB1 by RT-PCR from total RNA obtained from 3T3L1 cell culture after 16h in culture, with (+) and without (−) Reverse Transcriptase (RT). B) Detection of HMGB1 protein in different cell fractions by Western blot analysis. HSP60 is a cytoplasmic protein (Cy) (Antibody from Sigma-aldrich clone LK2) and H4 a nuclear protein (Nu) (Antibody from Santa-Cruz Biotechnology sc-10810).
(TIF)

Figure S3 **SW872 and 3T3L1 releases actively HMGB1 in cell culture media**. A) Quantification of HMGB1 secretion after renewal of cell culture media from SW872 cells in time dependent manner by Dot-Blot analysis. B) LDH assay on cell culture media from SW872 from A) according to manufacturer instructions (Sigma-aldrich). C) Quantification of HMGB1 and IL6 secretion after renewal of cell culture media from 3T3L1 cells in time dependent manner by respectively by Dot-Blot and ELISA assays.
(TIF)

Figure S4 **HMGB1 inhibition downregulates IL6 release**. A) Biochemical evaluation of HMGB1 purity by SDS-PAGE followed by Coomassie Blue staining or Western blotting with HMGB1 antibody (2F6 from Sigma-aldrich). B) Cytotoxicity evaluation of rHMGB1 on SW872 cell line using MTT assay according to manufacturer’s instructions (Sigma-aldrich). C) Impact of ethylpyruvate on HMGB1 secretion by Dot-Blot analysis.
(TIF)

Figure S5 **MCP1 (CCL2) and TNFα mRNA expression**. MCP1 and TNFα mRNA expression were assessed by RT-PCR from total RNA obtained from 3T3L1 cells after 12h of culture, with (+) and without (−) Reverse Transcriptase (RT).
(TIF)

Table S1 **List of primers used in this study for RT-PCR on 3T3L1 and SW872 RNA.**
(TIF)
