# Supplementary figures and images for: Soluble HMGB1 Is a Novel Adipokine Stimulating IL-6 Secretion through RAGE Receptor in SW872 Preadipocyte Cell Line: Contribution to Chronic Inflammation in Fat Tissue
Source: PLoS One. 2013 Sep 20;8(9):e76039. doi: 10.1371/journal.pone.0076039 (PMC3779194; doi:10.1371/journal.pone.0076039)

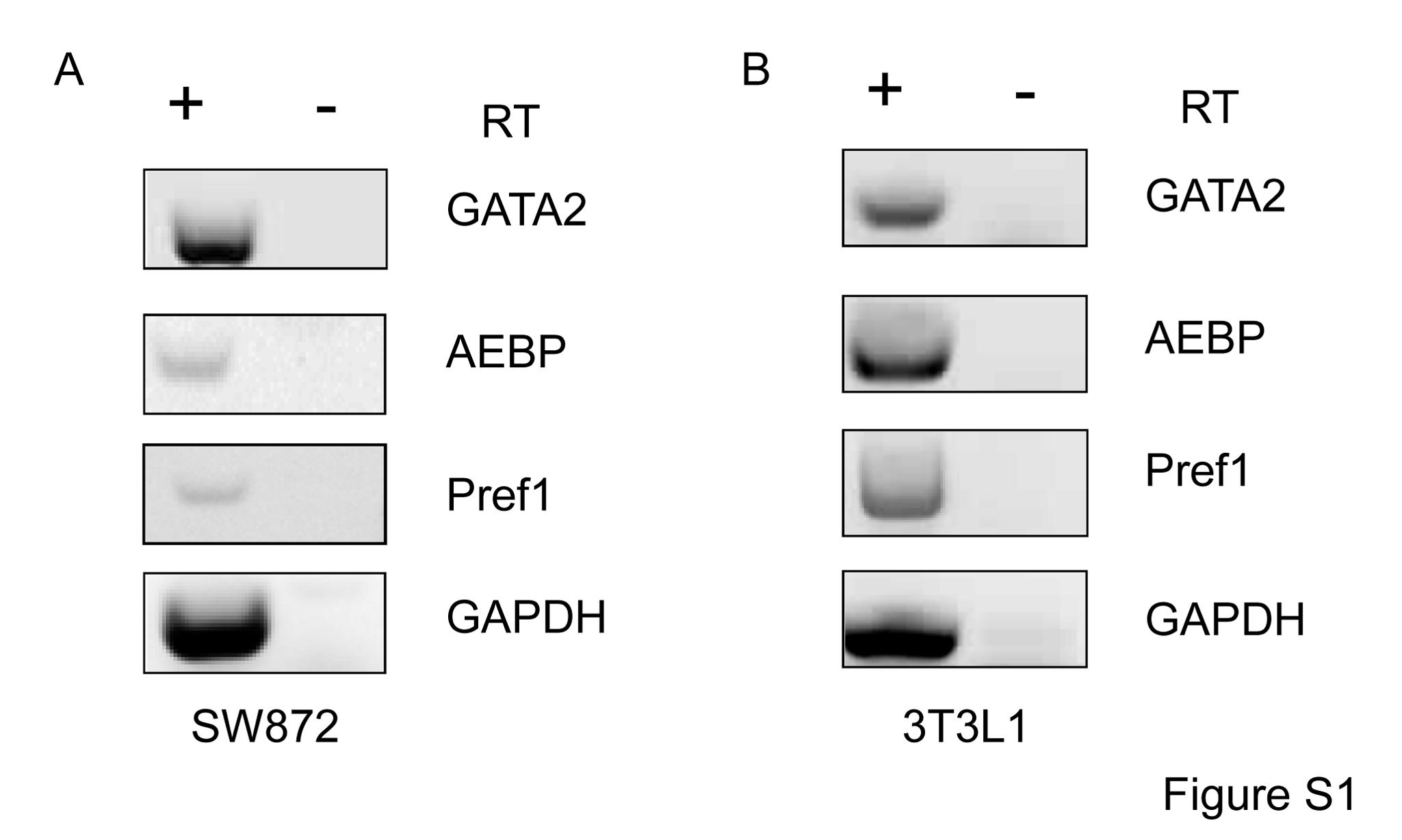

Supplement: Figure S1 — Preadipocyte markers expression in SW872 and 3T3L1 cell lines. Expression of pref-1, GATA2, AEBP by RT-PCR from total RNA obtained from SW872 cell after 12 h in culture (A) or from 3T3L1 after 16h in culture, with (+) and without (−) Reverse Transcriptase (RT). GAPDH served as housekeeping gene. (TIF) [file pone.0076039.s001.tif]

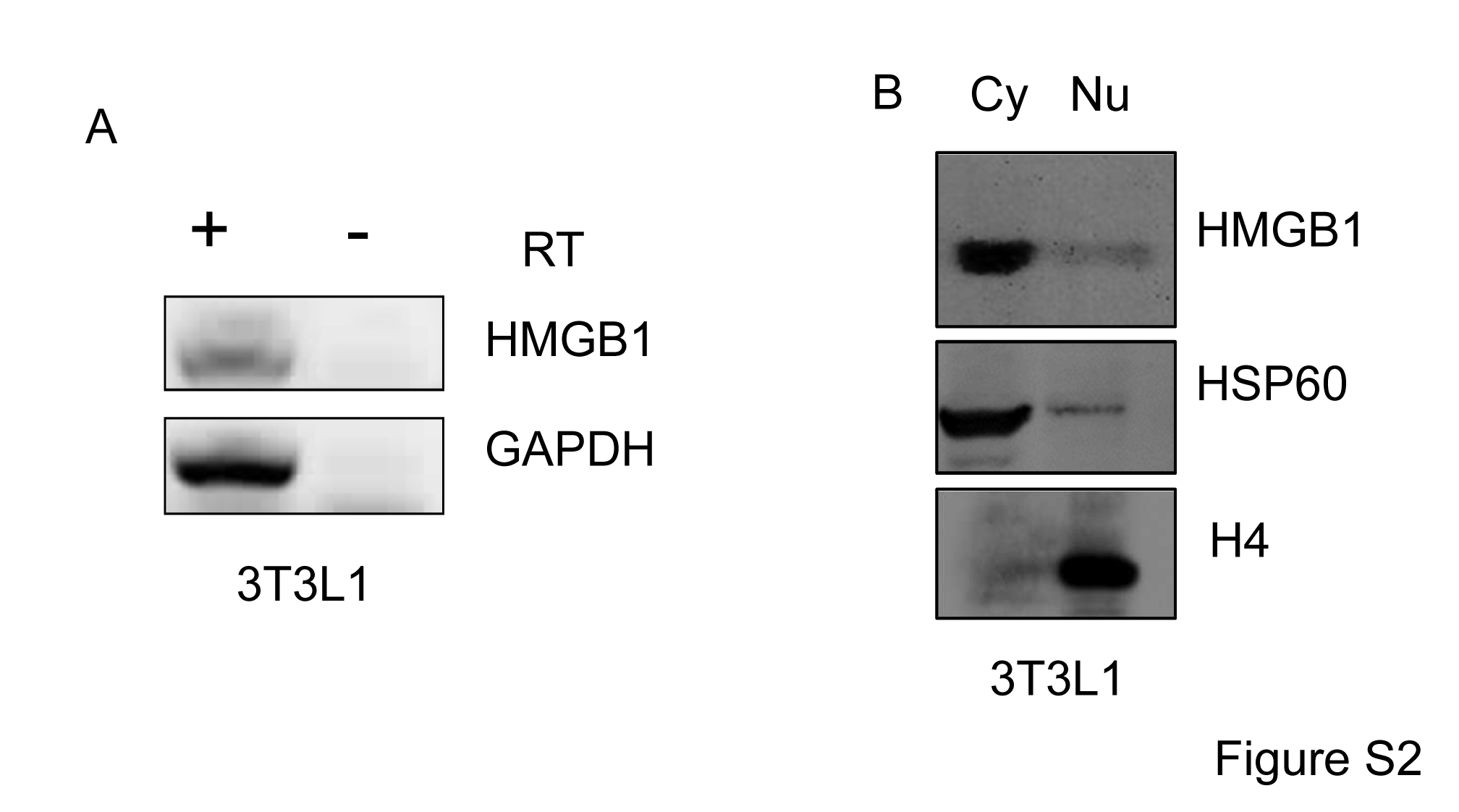

Supplement: Figure S2 — HMGB1 expression and localization in the human preadipocyte cell line 3T3L1. A) Expression of HMGB1 by RT-PCR from total RNA obtained from 3T3L1 cell culture after 16h in culture, with (+) and without (−) Reverse Transcriptase (RT). B) Detection of HMGB1 protein in different cell fractions by Western blot analysis. HSP60 is a cytoplasmic protein (Cy) (Antibody from Sigma-aldrich clone LK2) and H4 a nuclear protein (Nu) (Antibody from Santa-Cruz Biotechnology sc-10810). (TIF) [file pone.0076039.s002.tif]

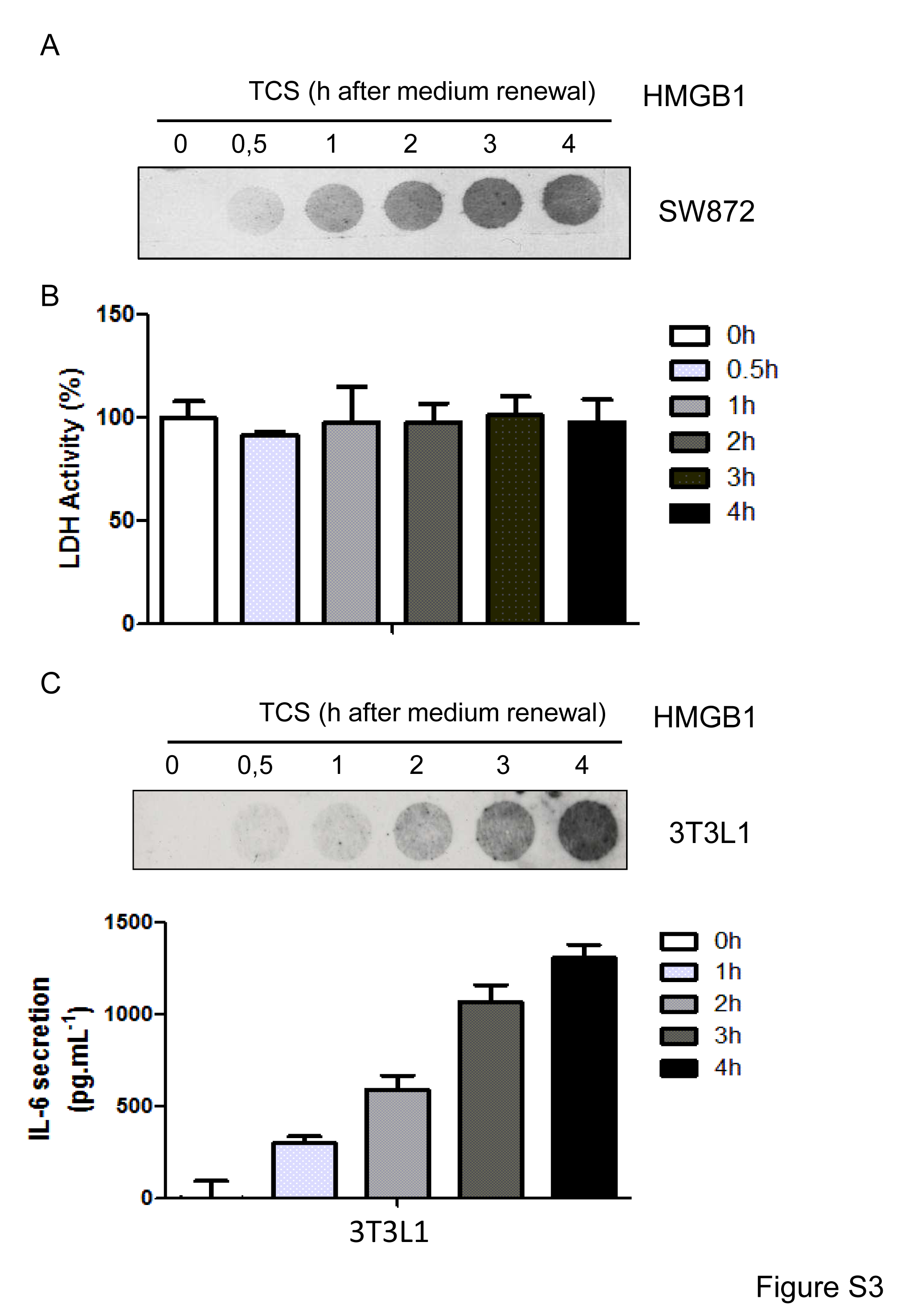

Supplement: Figure S3 — SW872 and 3T3L1 releases actively HMGB1 in cell culture media. A) Quantification of HMGB1 secretion after renewal of cell culture media from SW872 cells in time dependent manner by Dot-Blot analysis. B) LDH assay on cell culture media from SW872 from A) according to manufacturer instructions (Sigma-aldrich). C) Quantification of HMGB1 and IL6 secretion after renewal of cell culture media from 3T3L1 cells in time dependent manner by respectively by Dot-Blot and ELISA assays. (TIF) [file pone.0076039.s003.tif]

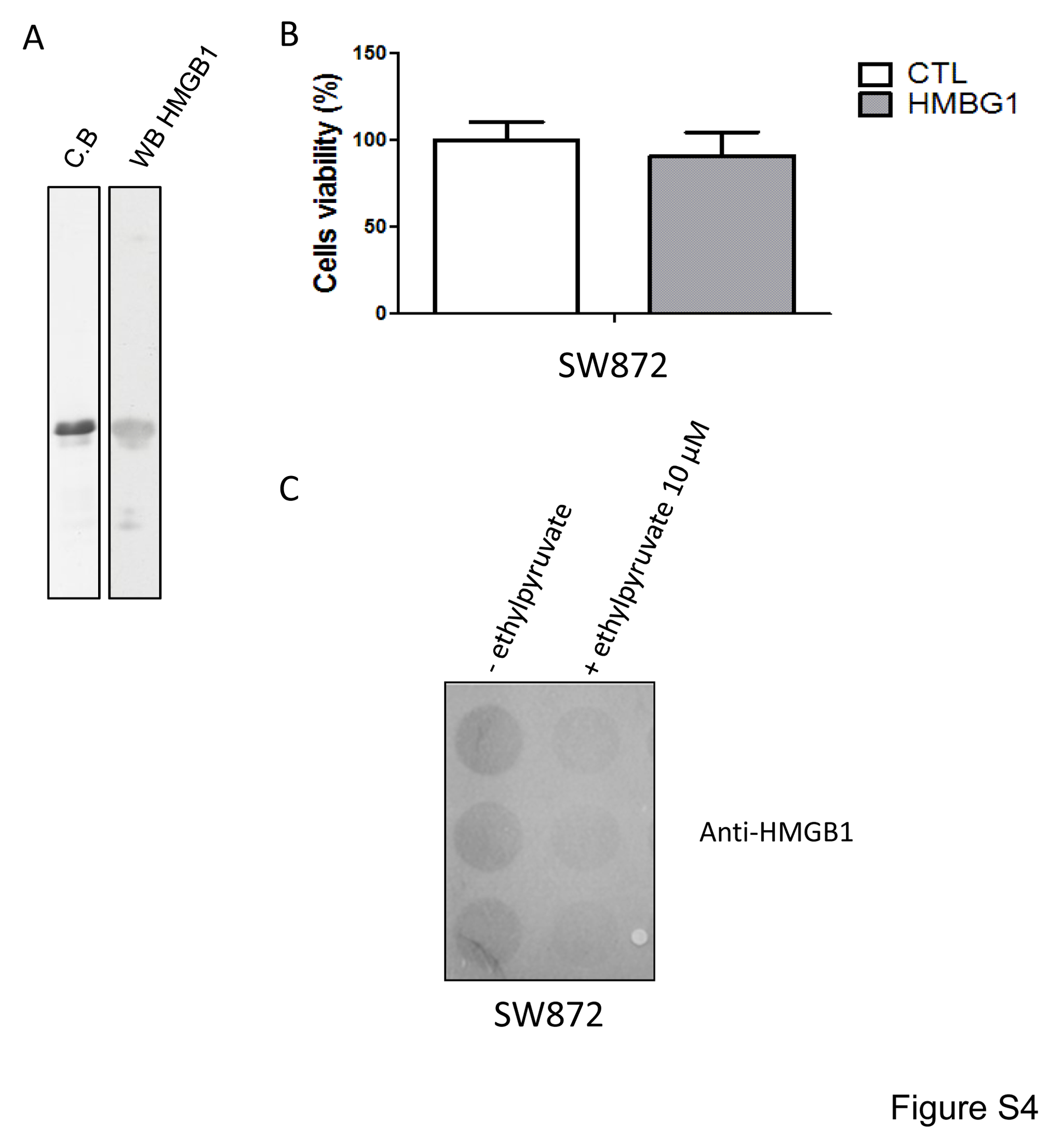

Supplement: Figure S4 — HMGB1 inhibition downregulates IL6 release. A) Biochemical evaluation of HMGB1 purity by SDS-PAGE followed by Coomassie Blue staining or Western blotting with HMGB1 antibody (2F6 from Sigma-aldrich). B) Cytotoxicity evaluation of rHMGB1 on SW872 cell line using MTT assay according to manufacturer’s instructions (Sigma-aldrich). C) Impact of ethylpyruvate on HMGB1 secretion by Dot-Blot analysis. (TIF) [file pone.0076039.s004.tif]

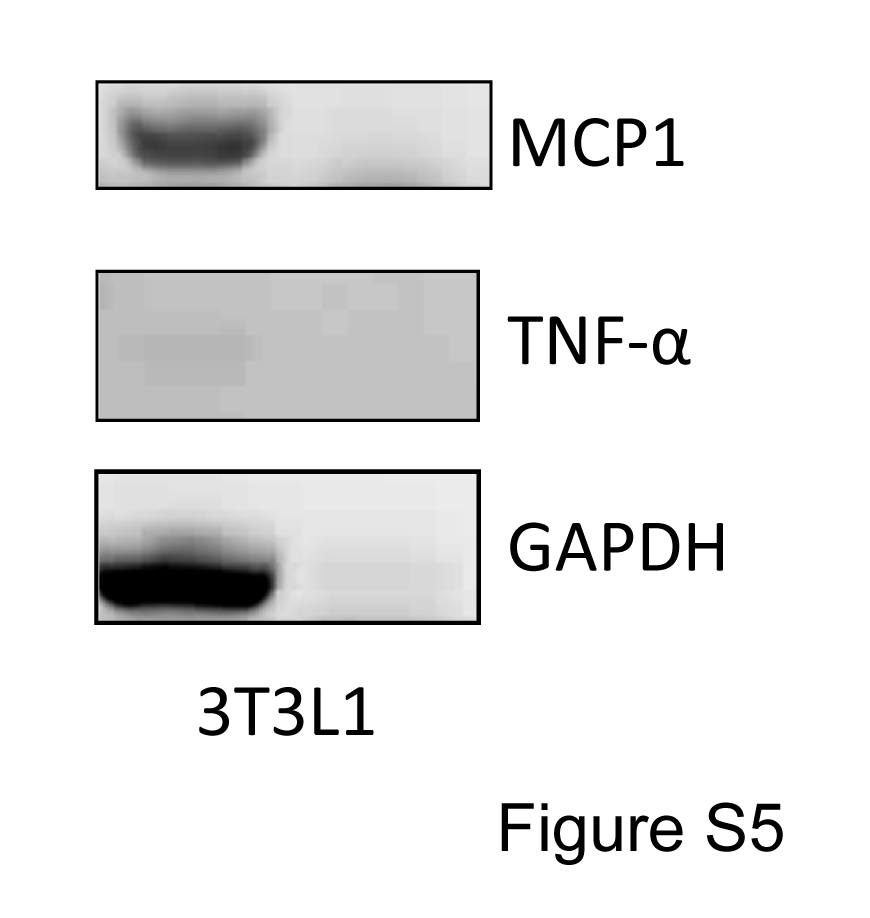

Supplement: Figure S5 — MCP1 (CCL2) and TNFα mRNA expression. MCP1 and TNFα mRNA expression were assessed by RT-PCR from total RNA obtained from 3T3L1 cells after 12h of culture, with (+) and without (−) Reverse Transcriptase (RT). (TIF) [file pone.0076039.s005.tif]

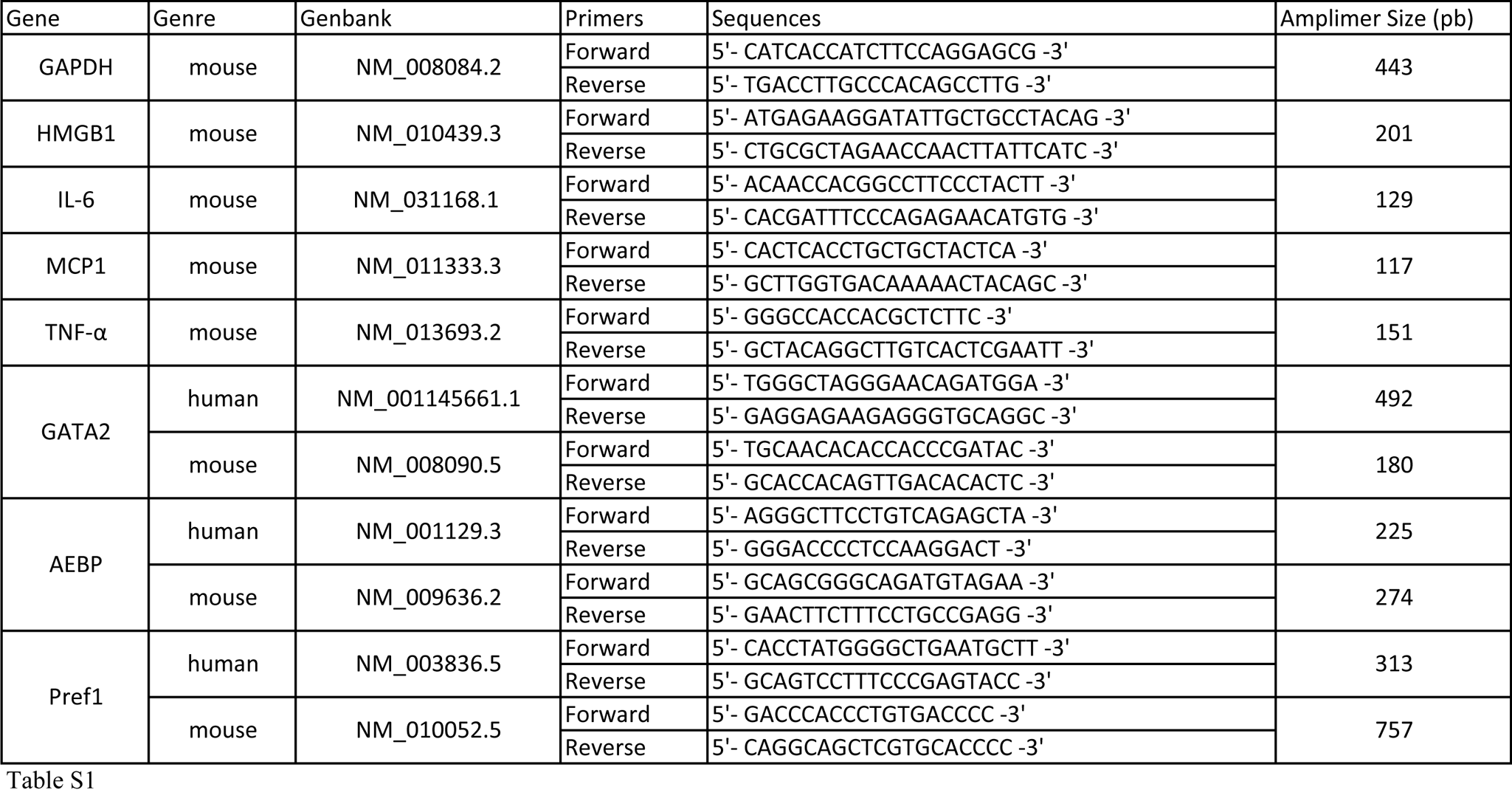

Supplement: Table S1 — List of primers used in this study for RT-PCR on 3T3L1 and SW872 RNA. (TIF) [file pone.0076039.s006.tif]
